# Non-vernalization Flowering and Seed Set of Cabbage Induced by Grafting Onto Radish Rootstocks

**DOI:** 10.3389/fpls.2018.01967

**Published:** 2019-01-10

**Authors:** Ko Motoki, Yu Kinoshita, Munetaka Hosokawa

**Affiliations:** ^1^Laboratory of Vegetable and Ornamental Horticulture, Graduate School of Agriculture, Kyoto University, Kyoto, Japan; ^2^Laboratory of Floriculture, Department of Agriculture, Kindai University, Nara, Japan

**Keywords:** cabbage, radish, grafting, vernalization, flowering, seed production, *FLOWERING LOCUS C*, *FLOWERING LOCUS T*

## Abstract

Cabbage (*Brassica oleracea* var. *capitata*) requires a long-term low-temperature exposure for floral induction, causing a delay in the breeding cycle. The objective of this study is to develop a method to induce flowering in cabbage without low-temperature treatment, using a grafting method. We conducted grafting experiments using two flower-induced Chinese kale cultivars (*B*. *oleracea* var. *alboglabra*) and seven radish cultivars/accessions as rootstocks and investigated the flowering response of grafted cabbage scions without low-temperature treatment. “Watanabe-seiko No.1” cabbage, when grafted onto the two Chinese kale cultivars, did not formed flower buds. Flowering was successfully induced in “Watanabe-seiko No.1” by grafting onto three out of the seven tested radish cultivars, and in “Kinkei No.201” and “Red cabbage” by grafting onto one tested radish cultivar. In “Watanabe-seiko No.1,” the earliest flower bud appearance was observed at 29 days after grafting. Seeds were also obtained from the three cabbage cultivars that flowered by grafting. Gene expression analysis of “Watanabe-seiko No.1” cabbage scions which formed flower buds by grafting, revealed high expression of the homolog of the floral integrator, *SUPPRESSOR OF OVEREXPRESSION OF CONSTANS 1* (*BoSOC1*), at the time of flower bud appearance. However, in the same leaf samples, we observed low expression of two homologs of florigen, *FLOWERING LOCUS T* (*BoFT.C2* and *BoFT.C6*). In addition, two homologs of the floral repressor *FLOWERING LOCUS C* (*BoFLC3* and *BoFLC4*), which are known to be down-regulated before flower bud differentiation in the vernalization pathway, were highly expressed, indicating that grafting onto radish induces cabbage flowering independently of the vernalization pathway. The expression level of the radish *FT* homolog (*RsFT*) in “Rat’s tail-G2,” which had highly induced flowering in the grafted cabbage scion, was higher than in the other radish cultivars. However, although “Rat’s tail-CH” effectively induced flowering in the cabbage scion, the expression of *RsFT* was low in this cultivar. In this study, floral induction of non-vernalized cabbage cannot be explained by the expression levels of *RsFT* in rootstock plants, alone. The flowering of non-vernalized cabbage would be induced by transmissible agents from rootstocks and not by the expression of cabbage *FT, BoFT*, from the scion itself.

## Introduction

Many Brassicaceae crops are induced to flower after exposure to low temperatures, a phenomenon known as vernalization. In the production of Brassicaceae crops whose vegetative organs, such as leaves and roots are harvested, flower stem development accompanying flowering leads to commercial value losses. Thus, late-bolting cultivars with strong vernalization requirements have been created for harvest in early spring. In some situations, vernalization requirements lead to a delay in the breeding cycle because of the increase in time required for flowering. For example, in cabbage (*Brassica oleracea* var. *capitata*), the delay in the breeding cycle due to the vernalization requirement is particularly remarkable, because cabbage is a plant-vernalization-type plant, which becomes sensitive to low temperatures only after developing to a certain size by expanding many true leaves, and also requires long-term low-temperature exposure for flowering. Furthermore, cabbage is a polycarpic plant, which has ability to turn back to vegetative growth after flowering; thus, reversion from reproductive growth to vegetative growth tends to occur when vernalization is insufficient (Ito and Saito, 1961). The vernalization requirement also limits regions suitable for seed production in plant-vernalization type plants such as cabbage. Research on floral induction by artificial low-temperature treatment (Wang et al., 2000) and gibberellin treatment (Nyarko et al., 2007) has been conducted with the intention of producing seed in tropical regions where cabbage seed production is difficult under natural conditions.

If we can develop a method enabling floral induction in cabbage without treatment at low temperatures, we can shorten the breeding cycle of cabbage and remove regional limitations for cabbage breeding and seeds production. Grafting is a known method for floral induction under non-inductive conditions in several kinds of plants (Chailakhyan, 1937; Zeevaart, 1976). Kagawa (1957) reported that he could induce cabbage flowering without low-temperature treatment by grafting it on a flowering radish (*Raphanus sativus*) rootstock. To our knowledge, this is the only report where cabbage has successfully flowered without low-temperature treatment. Interestingly, in this study by Kagawa (1957), when grafted onto flowering radish rootstocks, one in five cabbage scions flowered without low-temperature treatment; however, when grafted on flowering cabbage, no cabbage scions flowered. Kagawa discusses that this difference in floral induction of the grafted cabbages, may be caused by the difference in transmissibility of floral stimulating agents between radish and cabbage used as a rootstock. On the other hand, a different publication reported that cabbage did not flower at all when they were grafted onto flowering radish (Hamamoto and Yoshida, 2012). In our preliminary experiment, the percentage of cabbage that successfully flowered was dependent on the species and cultivars of rootstock plants. These results suggest that it may be possible to induce flowering of cabbage without low-temperature treatment by grafting onto flowering plants, but success may vary depending on the plant species and/or cultivars used as a rootstock.

Genes related to the vernalization flowering pathway have been identified in *Arabidopsis thaliana*, which belongs to the same plant family as cabbage, Brassicaceae. The floral repressor gene *FLC*, which acts as an integrator of the vernalization pathway, encodes a MADS-box protein that represses the expression of the floral promoter gene *FT* and floral integrator gene *SOC1* by directly binding to their first intron and promoter, respectively (Michaels and Amasino, 1999; Helliwell et al., 2006). Before low-temperature exposure, *FLC* is highly expressed and suppresses flowering. After the epigenetic silencing of *FLC* by prolonged low-temperature exposure, *FT, SOC1*, and other flowering-related genes are activated to promote flower bud differentiation (Bastow et al., 2004; Helliwell et al., 2006; Searle et al., 2006). The function of *FLC* is also conserved in *B*. *oleracea*, and *FLC* homologs are known to be involved in flowering time determination of several *B*. *oleracea* vegetables. *BoFLC4* (also known as *BoFLC2*) is one of the *FLC* homologs in *B*. *oleracea*, and was shown to be strongly associated with the vernalization requirement by QTL analysis in broccoli (*B*. *oleracea* var. *italica*) (Okazaki et al., 2007; Irwin et al., 2016), cabbage (*B*. *oleracea* var. *capitata*) (Okazaki et al., 2007), and cauliflower (*B*. *oleracea* var. *botrytis* L.) (Ridge et al., 2015). Meanwhile, *BoFLC3* was recently reported to be involved in curd induction variation in the subtropical broccoli breeding lines under the subtropical environment (Lin et al., 2018). *FT* homologs in *B*. *oleracea* have similar expression patterns to their ortholog in *A*. *thaliana* (Lin et al., 2005; Ridge et al., 2015; Irwin et al., 2016), and two *FT* loci (*BoFT.C2* and *BoFT.C6*) have been reported (Wang et al., 2012). *SOC1* homologs in *B*. *napus*, a closely related species of *B*. *oleracea*, have also similar expression patterns to their ortholog in *A*. *thaliana* (Guo et al., 2014). In radish, several transcriptome analysis suggested the involvement of the homologs of *FLC, FT*, and *SOC1* in the vernalization flowering pathway (Jung et al., 2016; Nie et al., 2016a,b). In several plant species, the FT protein is considered to be a major transmissible signal involved in floral induction by grafting (Corbesier et al., 2007; Lin et al., 2007; Tamaki et al., 2007; Notaguchi et al., 2008).

The final objective of this study is to develop a non-vernalization flowering method for cabbages, using a grafting method that enables a shortening of the breeding cycle and allows for seed production regardless of location or season. In this report, in order to identify rootstock plants suitable for our non-vernalization flowering method in cabbages, the flowering response of cabbages grafted onto radishes and Chinese kale (*B*. *oleracea* var. *alboglabra*) was investigated under controlled environmental conditions. We also discuss candidate genes important for the non-vernalization flowering of cabbage and the mechanism behind the differences in floral induction ability among rootstock plant cultivars.

## Materials and Methods

### Plant Materials and Growth Conditions

All plants were grown in a growth room maintained at 22 ± 2°C, PPFD 80 μmol m^-2^ s^-1^, irradiated with fluorescent lamps (NEC Lighting, Ltd., Japan), under long-day [16/8, light (L)/dark (D)] conditions. Japanese commercial cabbage cultivar, “Watanabe-seiko No.1” (Genebank Project, NARO, Japan, accession No. 25974), “Kinkei No.201” (Sakata Seed Corp., Japan), and “Red cabbage” (Nakahara Seed Product Co., Ltd., Japan) were used as scion plants for grafting experiment. It was previously reported that “Watanabe-seiko No.1” requires vernalization at 4–9°C for 6–7 weeks to induce flower bud differentiation (Ito and Saito, 1961). The two other cultivars require vernalization for flower bud differentiation as well. To reduce the effect of juvenility of the scion on its flowering response, cabbage seedlings grown for more than 10 weeks after sowing were used for collecting scions. The terminal shoots of the cabbage seedlings were pinched to elongate the lateral shoots, which were used as scions. For the grafting experiments using “Watanabe-seiko No.1” as a scion, two cultivars of Chinese kale “Kairan #1” (Tsurushin Seed Ltd., Japan) and “Kairan #2” (Kuragi Seed Ltd., Japan); four cultivars of radish for root vegetables (*R*. *sativus* L. var. *longipinnatus*) “Osaka-shijunichi” (Noguchi Seed, Japan), “Wakayama” (Noguchi Seed), “Shinshu-jidaikon” (Shinshu-sankyo Seed Co., Japan), and “Hayabutori-shogoin” (Takii Seed Co., Japan); and three accessions of radish for seed-pod vegetables (*R*. *sativus* L. var. *caudatus*) “Rat’s tail-CH” (Chiltern Seeds, United Kingdom), “Rat’s tail-G2” (Genebank Project, NARO, Japan, accession No. 76703), and “Rat’s tail-G4” (Genebank Project, NARO, Japan, accession No. 86212) were used as rootstocks (Table 1). These plants were chosen because flowering induction is easy; Chinese kale does not need low-temperature treatment for flowering, and radishes are seed vernalization plants that can be vernalized even at the seed stage. As a control, non-vernalized “Watanabe-seiko No.1” [“Watanabe-seiko No.1” (NVstock)] were also used as rootstock plants. For the low-temperature treatment of rootstocks, seeds were sown on wet filter paper and germinated in dark condition at 22°C for 1–2 days. The germinated seeds were incubated at 2°C in the dark for various, predefined periods of time (Table 1). The vernalized seeds were transplanted into 7.5 cm diameter plastic pots filled with granular rockwool (Nippon Rockwool Corp., Japan) and cultivated until bolting occurred. All plants were irrigated and fertilized from underneath the bottom of the pots using a half-strength nutrient solution (Enshi-shoho, formulated by the National Horticultural Research Station, Japan). For the grafting experiments using “Kinkei No.201” and “Red cabbage” scions, non-vernalized “Rat’s tail-CH” seedlings were grown in 6.0 or 7.5 cm plastic pots for use as rootstocks as described above.

**Table 1:** Basic features of rootstock plants used for grafting.

Species	Cultiver/Accession name	Origin	Seed vernalization treatment (days)	Days to grafting after sawing^z^	Number of leaves left on rootstock plant^z^	Estimated total area of leaves left on rootstock plant (cm^2^/plant)^z^
*B. oleracea* var. *capitata*	Watanabe-seiko No.1 (NVstock)^y^	Genebank project, NARO, Japan, accession No.25974	0	48.1 ± 3.2	4.6 ± 0.8	287.2 ± 67.8
*B. oleracea var. alboglabra*	Kairan #1	Tsurushin Seed Ltd., Japan	0	47.5 ± 8.6	6.4 ± 1.7	425.4 ± 114.3
	Kairan #2	Kuragi Seed Ltd., Japan	0	50.4 ± 1.7	7.0 ± 1.2	456.7 ± 51.6
*R. sativus* var. *longipinnatus*	Osaka-shijunichi	Noguchi Seed, Japan	6–8	34.6 ± 6.7	4.4 ± 1.3	226.8 ± 96.2
	Wakayama	Noguchi Seed, Japan	6–9	28.1 ± 4.1	4.3 ± 1.0	258.3 ± 109.6
	Shinshu-jidaikon	Shinshu-sankyo Seed Co., Japan	4–8	31.1 ± 6.8	5.0 ± 1.4	266.1 ± 88.2
	Hayabutori- shogoin	Takii Seed Co., Japan	18—21	23.0 ± 1.5	3.9 ± 0.9	149.2 ± 22.9
*R. sativus* var. *caudatus*	Rat’s tail-CH	Originally bought from Chiltern Seeds, United Kingdom, then seed propagated	0	41.2 ± 6.1	6.5 ± 2.2	528.5 ± 227.3
	Rat’s tail-G2 (2°C 0 day)	Genebank project, NARO, Japan, accession No. 76703	0	27.4 ± 0.55	5.2 ± 1.3	342.5 ± 148.4
	Rat’s tail-G2 (2°C 3 days)	Genebank project, NARO, Japan, accession No. 76703	3	19.75 ± 1.0	3.3 ± 0.5	133.1 ± 16.3
	Rat’s tail-G4	Genebank project, NARO, Japan, accession No. 86212	3	36.5 ± 7.1	5.9 ± 1.3	496.9 ± 277.4

*All the data of successfully grafted plants were used for the calculation of each item (n = 5–18). ^*z*^Mean ± SD, ^*y*^Non-vernalized rootstock as a grafting control*.

### Grafting of Cabbage Scions to Rootstock Plants

**FIGURE 1:**
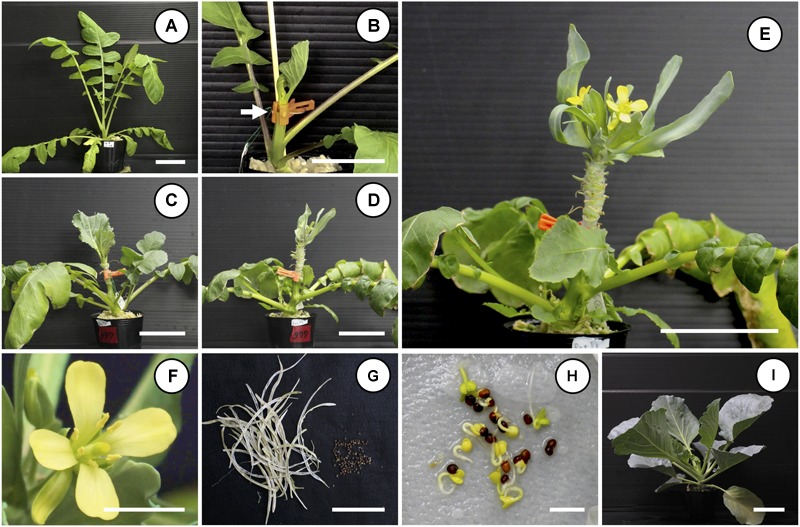
Non-vernalization flowering and seed production of “Watanabe-seiko No.1” cabbage by grafting onto a bolting radish rootstock. A shoot of “Watanabe-seiko No.1” with 2–3 leaves was grafted onto a stem of bolting radish rootstock. Grafted plants were grown in a growth room maintained at 22 ± 2°C, 16L/8D. Until flower bud appearance, the leaves of the grafted scion were continuously removed so that only 3–4 leaves remained. **(A)** Typical bolting radish rootstock used for grafting; the cultivar is “Hayabutori-shogoin.” **(B)** Rootstock and scion immediately after grafting. The white arrow indicates the grafted position. **(C–F)** Time-course pictures of non-vernalization flowering of “Watanabe-seiko No.1” grafted onto “Rat’s tail-CH,” **(C)** 25 DAG, **(D)** 53 DAG, **(E)** 57 DAG, and **(F)** close-up picture of **(E)**. **(G)** Seed pods and seeds produced by non-vernalization flowered “Watanabe-seiko No.1” grafted onto “Rat’s tail-CH.” Picture shows the whole seeds produced by one grafted scion. **(H)** Germination of the seeds of **(G)**. **(I)** Normal-grown seedlings of the seeds of **(G)**. Scale bars, 5 cm **(A–E,G,I)** and 1 cm **(F,H)**.

For radish rootstocks, seedlings that had bolted to a height of 5–8 cm from the top of the hypocotyl were used as rootstocks (Figure 1A). For two cultivars of Chinese kale rootstocks, seedlings at 5–8 weeks were used as rootstocks. For “Watanabe-seiko No.1” (NVstock), seedlings 6–7 weeks after sowing were used as rootstocks. The stem of the rootstock was cut at a height of 3–5 cm from the top of the hypocotyl, and the cabbage scion with 2–3 expanded leaves was grafted onto the stem of the rootstock by cleft grafting (Figure 1B). After grafting, the scion and a portion of the leaves of the rootstock plant were covered with a clear polyethylene bag to maintain high humidity, and the plants were grown under light conditions in the growth room. One to two weeks after grafting, once the scion and rootstock were fully connected, the polyethylene bag was removed. To promote translocation of assimilates from the rootstock to the scion, all the lateral shoots of the rootstock were removed and new leaves on the scion were removed with only 3–4 newly expanded leaves remaining, so that scion can keep sink activity.

### Self-Pollination of Cabbage Flowers

Cabbage scions that flowered after grafting were self-pollinated by CO_2_ treatment (Nakanishi and Hinata, 1975). Cabbage flowers were pollinated with self-pollen, and the inflorescence was immediately covered by two-layered clear polyethylene bags. Then, 120 mL of CO_2_ gas (equal to almost 20% of the volume of the polyethylene bag) was injected into the bag, with the opening tied. The polyethylene bags were removed 12–24 h after pollination.

### Measurements of the Growth and Flowering Parameters of the Rootstock and Scion

The number of leaves on the rootstock plants (*L*_n_) and the length (*L*_l_) and the width (*L*_w_) of the biggest leaf of the rootstocks were measured immediately after grafting. Then, the total leaf area of the rootstock (*L*_a_) was estimated using a preliminary calculated regression equation (Chinese kale: *L*_a_ = 0.2357 × *L*_n_ ×*L*_l_ ×*L*_w_ + 128.65, radish: *L*_a_ = 0.2407 ×*L*_n_ ×*L*_l_ ×*L*_w_ + 65.609, Supplementary Figure S1 and Supplementary Table S1). Plant growth continued until 60 days after grafting (DAG), and the appearance of flower buds was evaluated by daily visual inspection. On 60 DAG, the total number of expanded leaves of the scion (2 cm length or more) was counted, and scion flower bud differentiation was evaluated under a microscope if obvious flower buds were not visible. If the number of leaves of the scion at 60 DAG was less than 10, the grafting was regarded as a failure and the data were excluded from the calculation for the days to grafting after sawing, *L*_a_, number of expanded scion leaves, and percentage of scions with flower buds. The percentage of scions with flower buds was calculated as follows: (the number of individuals with differentiated flower buds at 60 DAG)/(the number of the successful grafts) × 100.

### Gene Expression Analysis

For gene expression analysis of flowering-related genes in the grafted rootstocks and scions, leaves were sampled as follows. The tip of third expanded leaf from the top of “Watanabe-seiko No.1” scions, which had flowered following grafting onto “Rat’s tail-CH” and “Rat’s tail-G2,” were sampled twice: at 7 DAG and within 1 week from flower bud appearance. As a control, the tip of third leaf from the top of “Watanabe-seiko No.1” scions grafted onto “Watanabe-seiko No.1” (NVstock) were sampled at 7 and 60 DAG. For the rootstock analysis, the tip of healthy and non-yellowing fresh leaves at the lowest position were sampled at 30 DAG. For comparison with “Watanabe-seiko No.1,” which had flowered by grafting, expression analysis of flowering-related genes was also performed on “Watanabe-seiko No.1,” which had been subjected to low-temperature treatment. The seedlings of “Watanabe-seiko No.1” were grown in the growth room for 10 weeks after sowing. Then, the seedlings were vernalized for 8 weeks in the growth cabinet, at a temperature of 6 ± 1°C, under LED light (660 nm:450 nm = 8:2) with an output of 90 μmol m^-2^ s^-1^ PPFD in long-day (16L/8D) conditions. After low-temperature treatment, the seedlings were returned to the growth room and continued to grow for 2 weeks. The eighth expanded leaf from the top was collected at the beginning and the end of the 8 weeks low-temperature treatment and at 2 weeks after returning to the growth room. All leaf samples were collected at 1 h before the end of the light period. Sampled leaves were immediately frozen with liquid nitrogen and stored at -80°C until RNA extraction.

Total RNA was extracted from leaves using Sepasol RNA I Super G (Nacalai Tesque, Kyoto, Japan), purified using a high-salt solution for precipitation (Takara Bio Inc., Ohtsu, Japan), and reverse transcribed with ReverTra Ace^®^ (Toyobo Co., Ltd., Osaka, Japan), following which 1 μL of 10-fold diluted reverse transcription (RT) product with pure water was used as a template for real-time RT-PCR. Real-time RT-PCR was performed using the THUNDERBIRD^®^ SYBR^®^ qPCR Mix (Toyobo Co., Ltd.) according to the manufacturer’s instructions using the LightCycler^®^ 480 system (Roche Diagnostics K.K., Tokyo, Japan). The real-time RT-PCR cycling was performed as follows: 95°C for 5 min, followed by 40 cycles at 95°C for 10 s, 60°C for 30 s, and 72°C for 30 s. Single-target product amplification was evaluated using a melting curve. All real-time RT-PCR primers used in this study are listed in Supplementary Table S2. The primer sequences were designed on the basis of structural gene sequences published in the NCBI databases^1^ and the report of Irwin et al. (2016). For primers analyzing individual homologous transcripts of *FLC* (*BoFLC3* and *BoFLC4*) and *FT* (*BoFT.C2* and *BoFT.C6*), particular caution was used to design primers for the different sequences between the two homologous genes. For the expression analysis of cabbage scion and vernalized “Watanabe-seiko No.1,” primer sets *BoFLC3, BoFLC4, BoFT.C2, BoFT.C6, BoSOC1*, and *BoActin* were used. For the expression analysis of “Watanabe-seiko No.1” and Chinese kale rootstocks, *BoFT.C6* and *BoActin* were used, whereas *RsFT* and *RsActin* were used for radish rootstocks.

### Sequence Analysis of *FT* Homologs

The PCR was performed using a primer set (Supplementary Table S2) that amplifies the full CDS of the *FT* homolog mRNA. RT products of total RNA extracted from the leaves of the rootstock plants were used as templates. The CDS was determined by direct sequencing of the amplification products using Sanger sequencing. The sequence data were deposited in DDBJ with accession numbers LC414370-LC414380.

## Results

### Initial Flowering Response of Rootstock Plants and Scion Growth After Grafting

Radish rootstocks bolted within approximately 20–40 days after sowing, with differences observed in the number of days until bolting among different cultivars (Table 1). The earlier the rootstock bolted, the smaller their leaf area tended to become, with the leaf area being the smallest in “Rat’s tail-G2” vernalized at 2°C for 3 days (133.1 ± 16.3 cm^2^) with the largest leaf area observed in “Rat’s tail-CH” (528.5 ± 227.3 cm^2^) (Table 1). Flower bud appearance was observed in some individuals of Chinese kale at the time of grafting (5th–8th weeks after sowing, data not shown). The leaf areas of the two Chinese kales were 425.4 ± 114.3 cm^2^ and 456.7 ± 51.6 cm^2^ for “Kairan #1” and “Kairan #2,” respectively. The leaf area of “Watanabe-seiko No.1” (NVstock) was 287.2 ± 67.8 cm^2^. The scion and the rootstock were successfully connected at 1–2 weeks after grafting, and the percentage for grafting success was approximately 70–80%, without large differences observed between rootstock cultivars (Table 2). The total number of expanded scion leaves at 60 DAG was on average 21.1 ± 2.0 to 32.1 ± 10.7 (Table 2). There was moderate positive relationship between the leaf area of the rootstock at 0 DAG and the number of expanded leaves of the scion at 60 DAG (*r* = 0.54, Supplementary Figure S2).

**Table 2:** Flowering response of “Watanabe-seiko No.1” cabbage grafted onto *B. oleracea* and *R. sativus* rootstocks.

Rootstock		Number of successful grafting (plant/plant)	Number of expanded leaves of scions^z^	Percentage of scions with flower bud (plant/plant)	Days to flower bud appearance^z,y^
Control	Non-grafted	8/8	–	0% (0/8)	–
	Watanabe-seiko No.1 (NVstock)	7/7	21.1 ± 2.0	0% (0/7)	–
*B. oleracea* var. *alboglabra*	Kairan #1	12/14	32.1 ± 10.7	0% (0/12)	–
	Kairan #2	13/18	27.1 ± 3.9	0% (0/13)	–
*R. sativus* var. *longipinnatus*	Osaka-shijunichi	17/18	23.8 ± 3.8	0% (0/17)	–
	Wakayama	17/19	21.7 ± 4.6	6% (1/17)	–
	Shinshu-jidaikon	18/18	23.2 ± 4.6	0% (0/18)	–
	Hayabutori-shogoin	18/22	22.9 ± 3.4	17% (3/18)	47
*R. sativus* var. *caudatus*	Rat’s tail-CH	12/16	29.3 ± 8.2	50% (6/12)	43.3 ± 10.1
	Rat’s tail-G2 (2°C 0 day)	5/8	23.6 ± 6.4	0% (0/5)	—
	Rat’s tail-G2 (2°C 3 days)	8/10	22.4 ± 2.2^x^	75% (6/8)	49.0 ± 6.7
	Rat’s tail-G4	13/19	21.2 ± 6.2	0% (0/13)	–

*Data were collected at 60 DAG. All the data of successfully grafted plants were used for the calculation of each item (n = 5–18), unless otherwise stated. ^*z*^Mean ± SD.*

*^*y*^The data of scions which formed visible flower bud within 60 DAG are presented (n = 1 for “Hayabutori-shogoin,” n = 6 for “Rat’s tail-CH,” and n = 5 for “Rat’s tail-G2” vernalized for 2°C, 3 days). ^*x*^Only the data for 5 of 8 plants are presented because of the lack of measurement*.

### Flowering of Grafted Scions and Seed Formation

**FIGURE 2:**
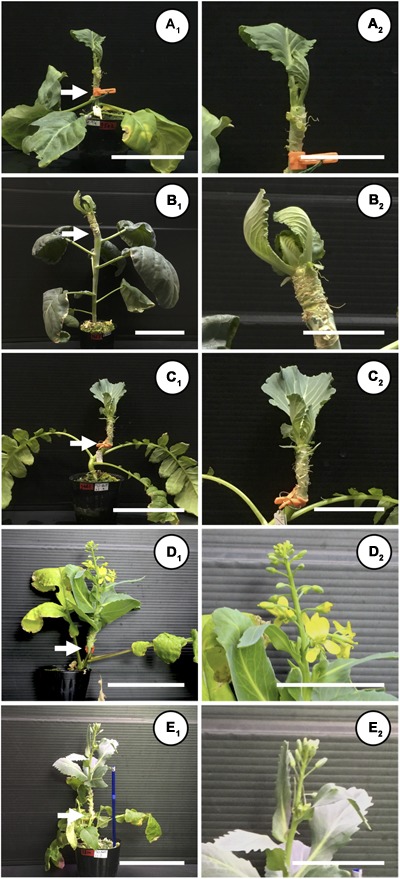
Different flowering responses of “Watanabe-seiko No.1” induced by different species and cultivars of rootstocks. A shoot of “Watanabe-seiko No.1” cabbage with 2–3 leaves was grafted onto a stem of a flowering-induced rootstock. Grafted plants were cultivated in a growth room maintained at 22 ± 2°C, 16L/8D. Until flower bud appearance, the leaves of grafted scions were continuously removed so that only 3–4 leaves remained. White arrow indicates graft union. **(A_1_,A_2_)** Control grafting where the scion was grafted onto a non-vernalized cabbage, “Watanabe-seiko No.1” at 60 DAG. The scion continued vegetative growth (**A_2_** is a close-up picture of **A_1_**, and the same applies for **B_1_–E_2_**). **(B_1_,B_2_)** A scion grafted onto Chinese kale “Kairan #1,” continuing vegetative growth at 60 DAG. **(C_1_,C_2_)** A scion grafted on radish “Shinshu-jidaikon,” continuing vegetative growth at 60 DAG. **(D_1_,D_2_)** A scion grafted onto radish “Rat’s tail-CH,” having several flower buds and opened flowers, at 47 DAG. **(E_1_,E_2_)** A scion grafted onto radish “Rat’s tail-G2” (2°C, 3 days), having several flower buds. Picture was taken at 60 DAG. Scale bars, 10 cm **(A_1_,B_1_,C_1_,D_1_,E_1_)** and 5 cm **(A_2_,B_2_,C_2_,D_2_,E_2_)**.

When “Watanabe-seiko No.1” was grafted onto non-vernalized “Watanabe-seiko No.1,” Chinese kales and some of the radish cultivars, the scions continued vegetative growth and did not form flower buds at 60 DAG (Table 2 and Figures 2A_1_–C_2_). On the other hand, a portion of the scions grafted onto radish cultivars “Wakayama” (1 out of 17), “Hayabutori-Shogoin” (3 out of 18), “Rat’s tail-CH” (6 out of 12), and “Rat’s tail-G2” (2°C, 3 days, 6 out of 8) formed flower buds (Table 2 and Figures 1C–E, 2D_1_,D_2_,E_1_,E_2_). In particular, the percentage of the scions with flower buds was high when “Rat’s tail-CH” and “Rat’s tail-G2” (2°C, 3 days) were used as the rootstock plants, with percentages of 50.0% and 75.0%, respectively (Table 2). The average number of days from grafting to flower bud appearance was 47 days, 43.3 ± 10.1 days, and 49.0 ± 6.7 days in “Hayabutori-Shogoin” (only one scion formed visible flower bud before 60 DAG), “Rat’s tail-CH,” and “Rat’s tail-G2” (2°C, 3 days), respectively (Table 2). The earliest flower bud appearance was observed at 29 DAG when “Rat’s tail-CH” was used as a rootstock plant. The scions flowered approximately 2–3 weeks after flower bud appearance (Supplementary Table S3), and most flowers formed normal floral organs with pistils and stamens (Figure 1F). The scions produced 2–3 inflorescences and finished blooming within approximately 1 month and returned to vegetative growth (Supplementary Figure S3).

We carried out self-pollination using the successfully flowered scions by CO_2_ treatment and obtained matured seeds roughly 80 days after pollination (Figure 1G). These seeds germinated and grew normally (Figures 1H,I). The “Kinkei No.201” and “Red cabbage” scions grafted onto “Rat’s tail-CH” also flowered and produced normal seeds (Supplementary Table S4 and Supplementary Figure S4).

### Expression Analysis of Flowering-Related Genes in Scions

**FIGURE 3:**
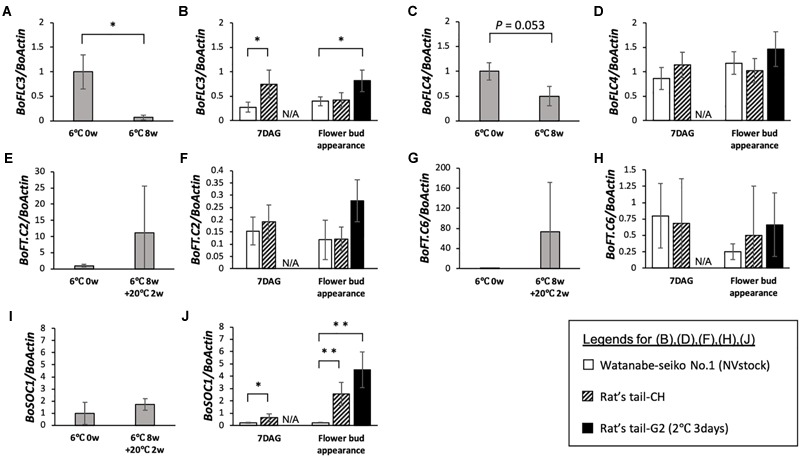
Expression analysis of flowering-related genes in the vernalized-flowered and non-vernalized graft-flowered “Watanabe-seiko No.1.” For each gene, expression levels normalized to the mean before vernalization (6°C 0 week) are shown. **(A,C,E,G,I)** Expression level of *BoFLC3, BoFLC4, BoFT.C2, BoFT.C6*, and *BoSOC1* in vernalized plants (*n* = 3 for each gene). **(B,D,F,H,J)** Expression level of *BoFLC3, BoFLC4, BoFT.C2, BoFT.C6*, and *BoSOC1* in control scions grafted onto “Watanabe-seiko No.1” (NVstock) (*n* = 4) and flowered scions grafted onto “Rat’s tail-CH” (*n* = 6) or “Rat’s tail-G2” (2°C, 3 days) (*n* = 4). The leaf samples collected at the point of “Flower bud appearance” were taken at 60 DAG for the control scions grafted onto “Watanabe-seiko No.1” (NVstock), and at within 1 week after flower bud appearance for the scions grafted onto “Rat’s tail-CH” or “Rat’s tail-G2” (2°C, 3 days). ^∗∗^ and ^∗^ indicate statistically significant differences (Student’s *t*-test, *P* < 0.01 and 0.05, respectively). N/A, data not available. Error bars are the standard deviations of the mean.

In 8 weeks vernalized “Watanabe-seiko No.1,” two out of three tested plants flowered within 1 month after the end of low temperature treatment. This partial flowering may be because the low temperature treatment in this study was not enough to fully induce flowering of this cabbage cultivar. On 8 weeks vernalized “Watanabe-seiko No.1,” the expression of two *FLC* homologous genes, *BoFLC3* and *BoFLC4*, decreased, relative to *BoActin*, to approximately 7 and 50% of the level from the start of treatment, respectively (Figures 3A,C). Conversely, in the graft-flowered scions, the expression levels of *BoFLC3* and *BoFLC4* were the same or higher than control plants grafted onto “Watanabe-seiko No.1” (NVstock), at both 7 DAG and flower bud appearance (Figures 3B,D). The expression level of *BoFT.C2* and *BoFT.C6* increased after low-temperature treatment with large variability (Figures 3E,G). This variation in the expression of *FT* homologs may reflect the inadequacy of low temperature treatment. In contrast, those *FT* homologs were very lowly expressed in all graft-flowered scions (Figures 3F,H). Further, the expression level of *BoSOC1* tended to increase after low-temperature treatment (Figure 3I). In the graft-flowered scions, the expression levels of *BoSOC1* significantly increased compared with the control grafts (Figure 3J).

### Expression and Sequence Analysis of *FT* Homologs of Rootstock Plants

In the previous studies, two *FT* homologs in *B. oleracea*, and one *FT* homolog in *R. sativus* have been reported (Wang et al., 2012; Nie et al., 2016b). To predict the candidate *FT* homologs which mainly act as transmissible floral signal in the Chines kale and radish rootstocks, we searched genes which have high homology with *Arabidopsis* FT protein (Accession No. AAF03936) in the NCBI reference sequence database of *B. oleracea* and *R. sativus* using BLAST program^2^. As a result, we could find three and two genes without insertion or deletion within any of the exons in *B. oleracea* and *R. sativus*, respectively (Supplementary Figure S5A). We also conducted BLAST genome search against reference genome database of *B. oleracea* (BOL reference Annotation Release 100) and *R. sativus* (Rs1.0 reference Annotation Release 100) and got the same result. Among those genes, two for *B. oleracea* and one for *R. sativus* were identical to the *FT* homologs reported in previous studies (XP_013619513/BoFT.C2, XP_013590834/BoFT.C6, XP_018470278/RsFT). Both of the other two proteins (XP_013635334/*B. oleracea*, XP_018460008/*R. sativus*) belonged to TSF clade by phylogenetic analysis (Supplementary Figure S5B). Although all of these genes were assumed to have FT-like function from their amino acid sequence, *BoFT.C6* and *RsFT* were chosen for expression analysis because they were expressed at much higher level than the other genes in the lowest leaf of Chinese kale and radish, in our preliminary experiment.

**FIGURE 4:**
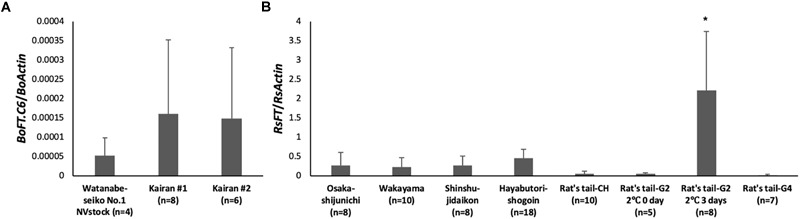
Expression analysis of *FT* homologs in leaves of rootstock plants at 30 DAG. The lowest leaves of the rootstock were sampled at 30 DAG, and the expression level of the *FT* homolog was determined by real-time RT-PCR. The number of plants investigated are shown in the graph for each cultivar (*n* = 4–18). **(A)** The expression level of *BoFT.C6* in “Watanabe-seiko No.1” and Chinese kale. *BoActin* were used as a reference gene. **(B)** The expression level of *RsFT* in radish cultivars. *RsActin* was used as a reference gene. ^∗^ indicates significant difference against other rootstocks by the Tukey HSD test (*P* < 0.001). Error bars are the standard deviations of the mean.

There were differences among the expression levels of *FT* homologs in each rootstock cultivar at 30 DAG (Figure 4). In the leaves of “Watanabe-seiko No.1” (NVstock), the *FT* homolog, *BoFT.C6*, was very lowly expressed. In Chinese kales, the *FT* expression levels were 2–3 times higher than “Watanabe-seiko No.1.” Expression level differed within radish cultivars. The expression level of *RsFT*, relative to the expression of *RsActin*, was the highest in “Rat’s tail-G2” (2°C, 3 days), and its expression level was significantly higher than all other cultivars. Conversely, the expression levels of *FT* homolog in “Rat’s tail-G2” (2°C, 0 day), “Rat’s tail-G4,” and “Rat’s tail-CH” were the lowest among radish cultivars.

Sequence analysis of the *FT* homolog CDS revealed several amino acid sequence polymorphisms within Chinese kale cultivars and between Chinese kale and radish (Supplementary Figure S6). However, no amino acid polymorphism was observed in the coding region among the radish cultivars used in this study.

## Discussion

### Flowering Mechanisms of Cabbage Scions Without Vernalization

**FIGURE 5:**
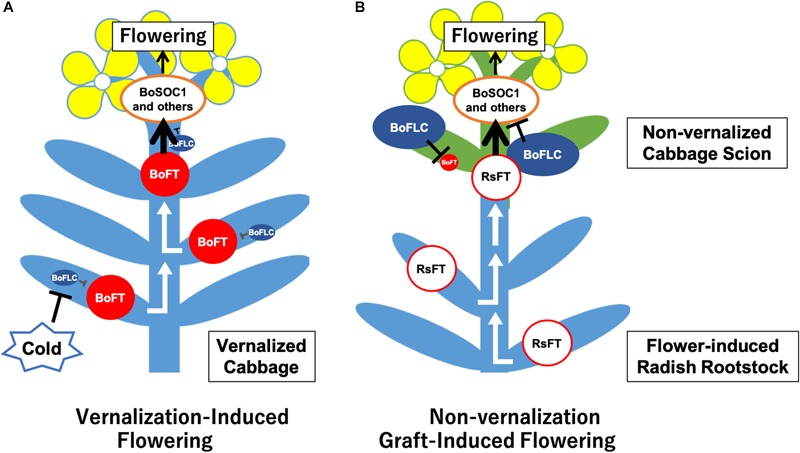
Assumed model of cabbage non-vernalization flowering induced by grafting onto radish rootstock in this study. **(A)** Vernalization induced cabbage flowering. After low-temperature treatment of cabbage seedlings, *BoFLCs* are down-regulated and *BoFTs* are up-regulated in the leaves and/or shoot apical meristem (SAM). Then, FT proteins are transported to the SAM, which up-regulates *BoSOC1s* and other flowering-related genes, inducing flower bud differentiation. **(B)** Non-vernalization cabbage flowering induced by grafting onto radish rootstock. In the cabbage scion that has not undergone low-temperature treatment, *BoFLCs* are highly expressed and repress *BoFTs* expression. The RsFT protein and/or another transmissible signals are transported from the radish rootstock to the cabbage scion, up-regulating *BoSOC1s* and other flowering-related genes in the cabbage scion SAM, inducing flower bud differentiation by bypassing the flowering repression created by BoFLCs.

We could successfully induce flowering in cabbage scions without low-temperature treatment by grafting them onto radish rootstocks (Figures 1C–E, 2D_1_,E_1_, Table 2, Supplementary Figures S4A,D and Supplementary Table S4). Expression analysis of flowering-related genes was conducted to investigate the mechanism of cabbage scion flowering without vernalization. Low-temperature treatment resulted in decreased expression of *FLC* homologs and increased expression of *FT* and *SOC1* homologs in “Watanabe-seiko No.1” (Figures 3A,C,E,G,I). Therefore, the expression pattern of *FLC, FT*, and *SOC1* observed in *A. thaliana* appears to be conserved in “Watanabe-seiko No.1.” Conversely, in “Watanabe-seiko No.1” plants that flowered after grafting, the expression levels of *FLC* homologs were the same as or higher than that of the control graft (Figures 3B,D). Moreover, since *FT* homologs are marginally expressed in graft-flowered scions (Figures 3F,H), flower bud differentiation within the cabbage scion would not be induced by transcribed *FT* originating in the scion. The expression level of *SOC1* homologs was significantly higher in graft-flowered “Watanabe-seiko No.1” than in the control graft (Figure 3J). In several plant species, the FT protein has been shown to be a major constituent of florigen, which is synthesized in leaves and transported to the shoot apex, leading to floral transition (Corbesier et al., 2007; Lin et al., 2007; Tamaki et al., 2007; Notaguchi et al., 2008). It is also known that *SOC1* is up-regulated via *FT* expression (Yoo et al., 2005). Considering that endogenous *FT* homologs are minimally expressed in the scion, *SOC1* homologs would be activated by mobile signals transmitted from the rootstocks. In *FLC*-mediated late-flowering *Arabidopsis, FT* overexpression activated *SOC1* expression and strongly suppressed the late-flowering phenotype; however, it did not affect *FLC* mRNA level (Michaels et al., 2005). On the basis of this evidence, we assumed that in the graft-induced flowering of cabbage without vernalization, exogenous mobile signals like the FT protein are transported from the rootstock, inducing flowering of the cabbage by bypassing the flowering suppression created by FLC by directly activating *SOC1* and the other flowering-related genes expression (Figure 5).

“Watanabe-seiko No.1,” which flowered after grafting, developed a few inflorescences and then returned to vegetative growth (Supplementary Figure S3). This suggests that the flowering signals transported from the rootstock were not self-amplifying, and thus the floral induction of the scion would occur in a dose-dependent manner. This reversion from reproductive growth to vegetative growth in a relatively short period could support the above hypothetical molecular mechanism of graft-induced flowering of cabbage.

### Why Flower Bud Differentiation in Cabbage Scions Varied Depending on Rootstock Genera or Cultivars

To identify rootstocks that can induce flowering, we investigated factors of the rootstock plants related to floral induction of the scion. From the results of gene expression analysis of the graft-flowered cabbage scions, the FT protein is the hypothesized candidate for a causal mobile signal transported from the rootstock. Notaguchi et al. (2008) reported that in a grafting experiment using the *A. thaliana ft* mutant as a recipient, the higher the *FT* expression level of donor plant was, the more likely the scion was to flower. We hypothesized that the variation in successful flower bud formation was due to the difference in expression of FT proteins in the leaves of the rootstocks. Therefore, we measured the expression levels of *FT* mRNA in the rootstocks at 30 DAG, when the scion and rootstock had already been well connected and translocation between them would be actively occurring. Then, we examined the relationship between the flower bud differentiation rate of the scion and the *FT* expression level of the rootstock.

We found that the expression level of the *FT* homolog tended to be high in the rootstock plants that had induced flower bud differentiation in the scion (Figure 4). The *FT* homolog was minimally expressed in “Watanabe-seiko No.1” (NVstock). In addition, in the two cultivars of Chinese kale that did not induce flower bud differentiation, the expression level of *FT* homolog was approximately 30 times lower than “Watanabe-seiko No.1” that had been vernalized for 8 weeks (Figures 3G, 4A). Considering that the Chinese kale cultivars used in this study do not require low-temperature treatment for flowering, it seemed that the Chinese kales may be induced to flower by a relatively small amount of FT protein or that the contribution of FT protein expressed in the lower leaves is small. The expression level of the *FT* homolog in “Rat’s tail-G2” (2°C, 3 days), which induced the highest percentage of cabbage scions to flower, was significantly higher than all other rootstocks (Figure 4B). Since there was no significant difference in the expression level of *RsFT* by the position within a leaf in the preliminary experiment (Supplementary Figure S7), the leaf tip we used for the analysis would represent the trend of *RsFT* expression level of the whole leaf. We presume that the total amount of *RsFT* mRNA/protein expressed in a whole plant would also be the highest in “Rat’s tail-G2” (2°C, 3 days), considering the relatively small difference in the total leaf area between radish cultivars (Table 1). Moreover, the grafted “Rat’s tail-G2” (including both 2°C, 0 day; and 2°C, 3 days vernalized rootstocks) showed a strong negative correlation between the expression level of the rootstock *FT* homolog and the number of days to flower bud appearance in cabbage scions (*r* = -0.76, Supplementary Figure S8). These results indicate the contribution of rootstock leaf FT expression on flowering in the cabbage scion.

Conversely, the *FT* homolog was lowly expressed in “Rat’s tail-CH,” which highly induced scion flower bud differentiation (Figure 4B). Because there were no amino acid polymorphisms in the *FT* homolog among the radish cultivars used in this study (Supplementary Figure S6), the differences in the mobility or function of the FT protein may not be the cause of the high percentage of successful flower bud initiation of the cabbage grafted onto “Rat’s tail-CH.” Thus, it was implied that mobile factors other than the FT protein may be involved in the flowering of the cabbage grafted onto “Rat’s tail-CH.” Of course, we also should take into consideration that the *FT* mRNA expression level does not always reflect the protein expression level (Kim et al., 2016) and that the accumulation of the FT protein at the shoot apex can be affected by the efficiency of transport through phloem tissue (Liu et al., 2012). Different results may be obtained if the accumulation of the FT protein in the scion is investigated, and further investigation is needed to identify the contributing factors of the rootstocks responsible for the differences in floral induction.

### Future Applications in Agriculture

A method enabling floral induction of cabbage without low-temperature treatment would be a useful tool in cabbage breeding and seed production, by shortening the breeding cycle and removing geographical constraints on seed production. Three methods of inducing flowering in plants under non-inductive conditions have been reported in previous studies, including exogenous plant hormone treatment (Lang, 1957; Trusov and Botella, 2006), transient expression or silencing of flowering-related genes (Lin et al., 2007; Aìlvarez-Venegas et al., 2010; Yamagishi et al., 2011), and grafting (Chailakhyan, 1937; Zeevaart, 1976). Although some plant hormones can induce flowering in several plant species under non-vernalized condition (Lang, 1957; Trusov and Botella, 2006), there are no reports in which a plant hormone can substitute for low-temperature treatment in the flowering of cabbage. For example, gibberellin has been known to induce flowering in several long-day and vernalization-requiring plants (Lang, 1957); however, the exogenous application of gibberellin did not induce flowering in cabbage without low-temperature treatment (Hamano et al., 2002; Nyarko et al., 2007). Transient expression or silencing of flowering-related genes using viral vectors is a powerful tool for inducing early flowering under non-inductive conditions (Lin et al., 2007; Aìlvarez-Venegas et al., 2010; Yamagishi et al., 2011). In winter canola (*B*. *napus*), a closely related species of cabbage, the silencing of *FLC* homologs (*BnFLC*) by RNA interference using a viral vector enabled flowering without cold treatment (Aìlvarez-Venegas et al., 2010). However, in cabbage, a transient expression method using a viral vector has not been established. This method also requires careful attention to prevent the escape of the recombinant virus into the external environment. Thus, presently, it is not practical to use viral vectors as an early flowering technique in cabbage.

Alternatively, grafting is a simple method that in this study successfully induced flowering in cabbage without low-temperature treatment, in accordance with the previous report (Kagawa, 1957). Seeds were obtained from the flowered scions, and the seeds developed normally into seedlings (Figures 1G–I and Supplementary Figures S4B,C,E,F), confirming the potential use of this flowering method to breed and produce cabbage seed. It was reported that “Watanabe-seiko No.1” cabbage requires 6–7 weeks vernalization at 4–9°C to induce flower bud differentiation (Ito and Saito, 1961). When “Watanabe-seiko No.1” was grafted onto “Rat’s tail-CH” in this study, the earliest flower bud appearance occurred at 29 DAG, suggesting that the grafting method would shorten the period needed to obtain cabbage seeds, when compared with low-temperature treatment. Another advantage of the grafting method is that less space is needed for cultivation, considering that a small scion (about 4–6 cm long) could be induced to flower by grafting in this study, in contrast to the many true leaves that need to expand in order for a cabbage to be induced to flower by low-temperature treatment (Ito and Saito, 1961). This will be an advantage of cabbage floral induction in a closed environment, enabling stable and rapid seed production year-round. As mentioned above, the floral induction ability among the rootstock plants varied, depending on their genus or cultivar, and the expression of the *FT* homolog in the rootstock leaves was hypothesized to be one of the contributing factors. Identifying and breeding rootstock plants that can strongly induce cabbage flowering after grafting will be important to the future success of our grafting method in commercial applications. Interestingly, both of the two radish cultivars, “Rat’s tail-CH” and “Rat’s tail-G2,” which successfully induced cabbage flowering at a high rate, are seed-pod radishes (*R*. *sativus* L. var. *caudatus*), which originally distribute in tropical areas. This suggests that plants originating in tropical areas that do not require vernalization for floral induction may have the ability to strongly induce flowering by grafting, possibly because they maintain high expression of transmissible floral stimulating agents, like FT, under warm conditions. Although cabbage scions could produce normal seeds without low-temperature treatment after grafting onto a “Rat’s tail-CH” rootstock (Figures 1G–I and Supplementary Figures S4B,C,E,F), there may be other rootstock plants that can more efficiently induce cabbage flowering, within radish cultivars or in other plant species.

## Conclusion

In this study, we successfully induced flowering and seed set of several cabbage cultivars without low-temperature treatment by grafting onto radish rootstocks under a controlled environment. Our results demonstrate the usefulness of this graft-induced flowering method for shortening the cabbage breeding cycle and its potential seed production in tropical areas. Interestingly, there was a significant difference in the flowering response of the cabbage scion depending on which radish cultivars were used as rootstocks. The expression level of the *FT* homolog in the rootstock plant appears related to cabbage scion floral induction. Further investigation will make it possible to identify the factors within the rootstock that contribute to flowering in the cabbage scion. This information will be useful for screening and breeding rootstocks that strongly induce flowering of the scion by grafting and thus promoting the practical use of this graft-inducing flowering method not only for cabbage but also other Brassicaceae crops.

## Author Contributions

KM contributed to data collection, data analysis, and writing of the manuscript. YK contributed to data analysis, data interpretation, drafting the manuscript, and final approval of the version. MH contributed to the conception and design of the work, data analysis, data interpretation, writing the manuscript, and final approval of the version.

## Conflict of Interest Statement

The authors declare that the research was conducted in the absence of any commercial or financial relationships that could be construed as a potential conflict of interest.

## Footnotes

1 https://www.ncbi.nlm.nih.gov

2 http://www.ncbi.nlm.nih.gov/BLAST/

## Abbreviations

CDS: coding sequence
DAG: days after grafting
*FLC*: *FLOWERING LOCUS C*
*FT*: *FLOWERING LOCUS T*
PPFD: photosynthetic photon flux density
SAM: shoot apical meristem
*SOC1*: *SUPPRESSOR OF OVEREXPRESSION OF CONSTANS 1*
