# Supplementary material for: Non-vernalization Flowering and Seed Set of Cabbage Induced by Grafting Onto Radish Rootstocks
Source: Front Plant Sci. 2019 Jan 10;9:1967. doi: 10.3389/fpls.2018.01967 (PMC6335391; doi:10.3389/fpls.2018.01967)
Supplement: Supplementary file 1 [file Data_Sheet_1.docx]

Supplementary Material

**Non-vernalization Flowering and Seed Set of Cabbage Induced by Grafting onto Radish Rootstocks**

Ko Motoki^1^, Yu Kinoshita^1^ and Munetaka Hosokawa^1,2^**^*^**

*** CORRESPONDENCE:** Dr. Munetaka Hosokawa: [mune@nara.kindai.ac.jp](mailto:mune@nara.kindai.ac.jp)

**
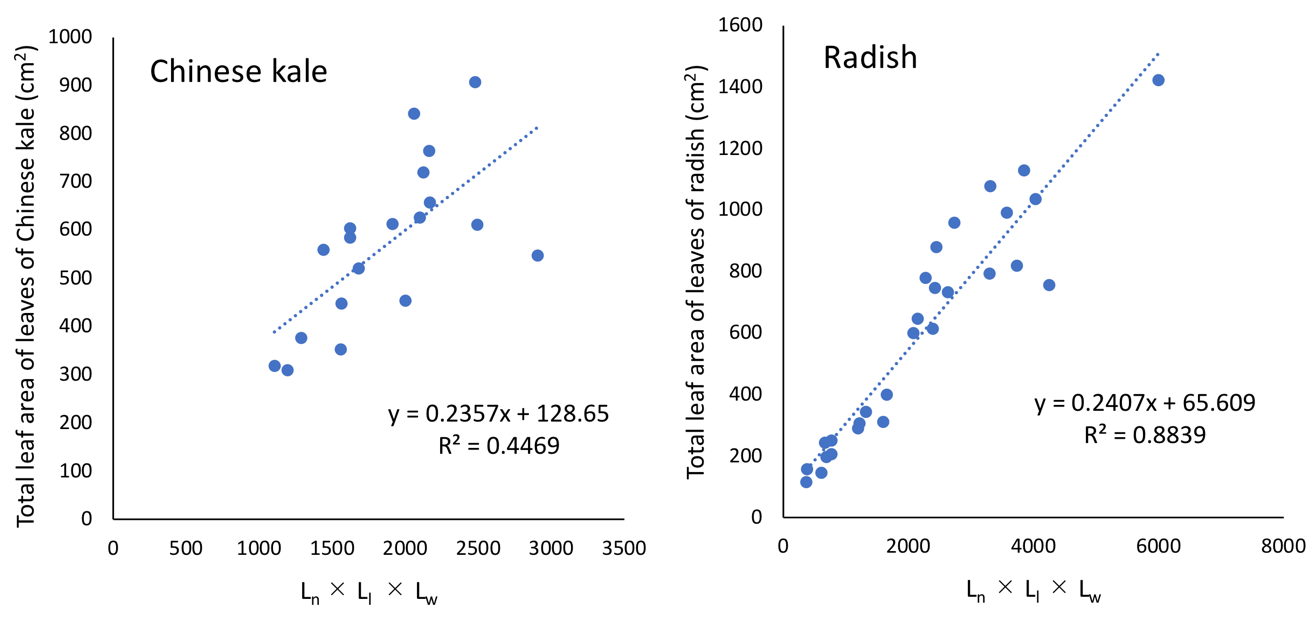
**

**Supplementary Figure 1.** The relationships between leaf area and leaf parameters of rootstock plants. Bolting Chinese kale (*n* = 19) and radish (*n* = 28) were prepared with the stems cut according to the grafting method described in the Materials and Methods. The numbers of leaves remaining on the rootstock plants (L_n_) were counted, and the length (L_l_) and width (L_w_) of the largest leaf were also measured. The total leaf areas of leaves remaining on the rootstock plants were measured by using LI-3100C Area Meter (LI-COR, Inc., USA). The leaf areas of each bolting rootstock plant were plotted against the product of L_n_ × L_l_ × L_w_. Data were analyzed using regression analysis in which the dependent variable (Y) was the combined total leaf area of leaves remaining on the rootstock plant, and the independent variable (X) was the product of L_n_ ×  L_l_ × L_w_. The results were fitted by the regression equation Y = aX + b in which a and b are the regression coefficients.

**
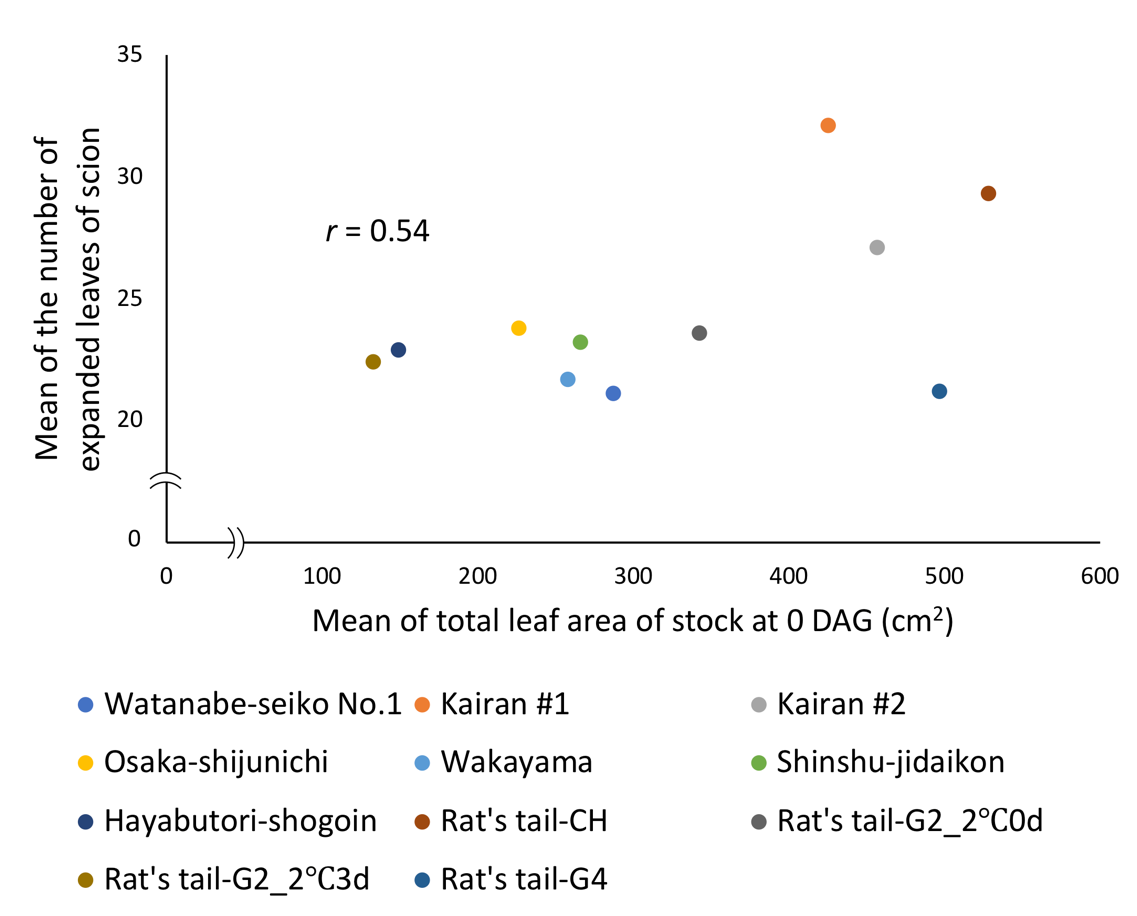
**

**Supplementary Figure 2.** The relationships between total leaf area of rootstock plants at 0 DAG and the number of expanded leaves of scions.

**
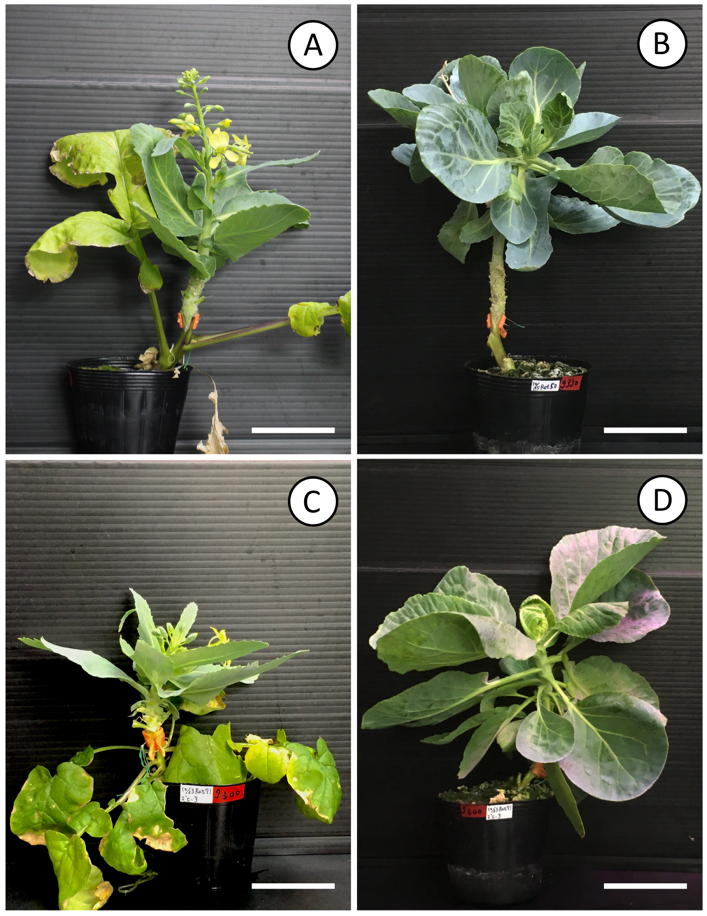
**

**Supplementary Figure 3.** Reversion to vegetative growth after grafting-induced non-vernalization flowering. **(A)** Non-vernalization flowering of “Watanabe-seiko No.1” grafted onto “Rat's tail-CH” (47 DAG). **(B)** Reversion to vegetative growth of (A) (140 DAG). **(C)** Non-vernalization flowering of “Watanabe-seiko No.1” grafted onto “Rat's tail-G2” (54 DAG). **(D)** Reversion to vegetative growth of (C) (145 DAG). Scale bars, 5 cm.

**
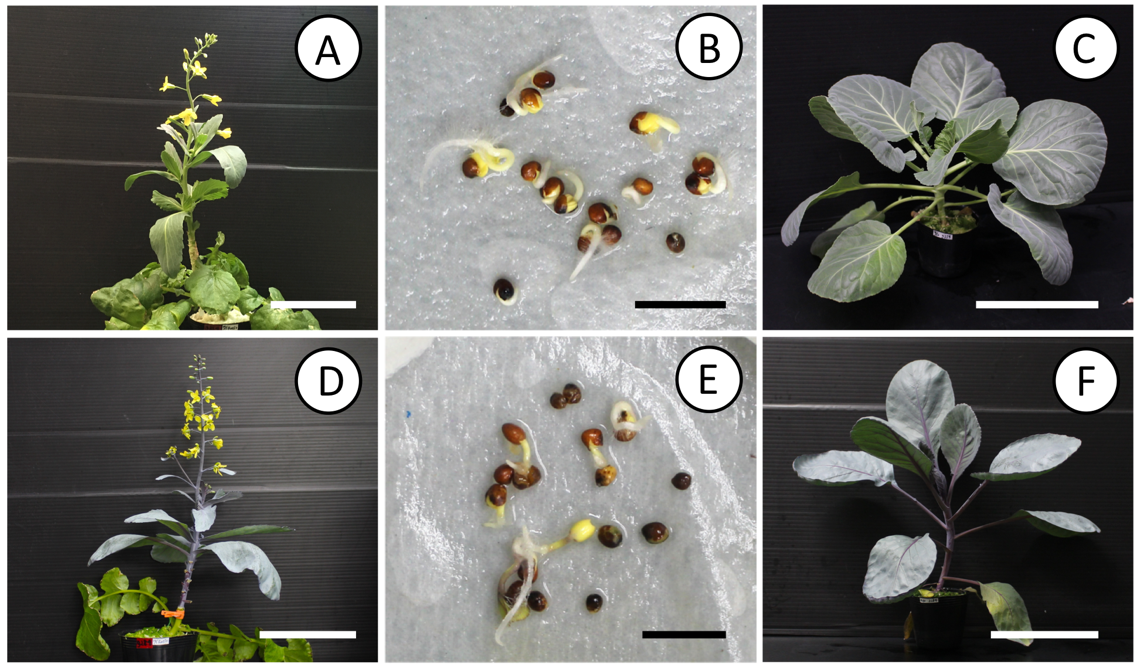
**

**Supplementary Figure 4.** Non-vernalization flowering and seed production of commercial cabbage cultivars “Kinkei No.201” and “Red cabbage” after grafting onto the “Rat's tail-CH” radish. Cabbage shoots grown in growth room (22 ± 2°C, 16L/8D) were grafted onto a stem of bolting radish rootstock. Grafted plants were grown in the same growth room. Until flower bud appearance, the leaves of grafted scions were continuously removed so that only 3–4 leaves remained. **(A)** Non-vernalization flowering of “Kinkei No.201” (61DAG). **(B)** Germination of the seeds produced by the grafted shoot of (A). **(C)** Normal-grown seedlings from the seeds of (B). **(D)** Non-vernalization induced flowering in “Red cabbage” (52 DAG). **(E)** Germination of the seeds produced by the grafted shoot of (D). **(F)** Normal-grown seedlings of the seeds of (E). Scale bars, 10 cm (A, C, D, and F) and 1 cm (B and E).


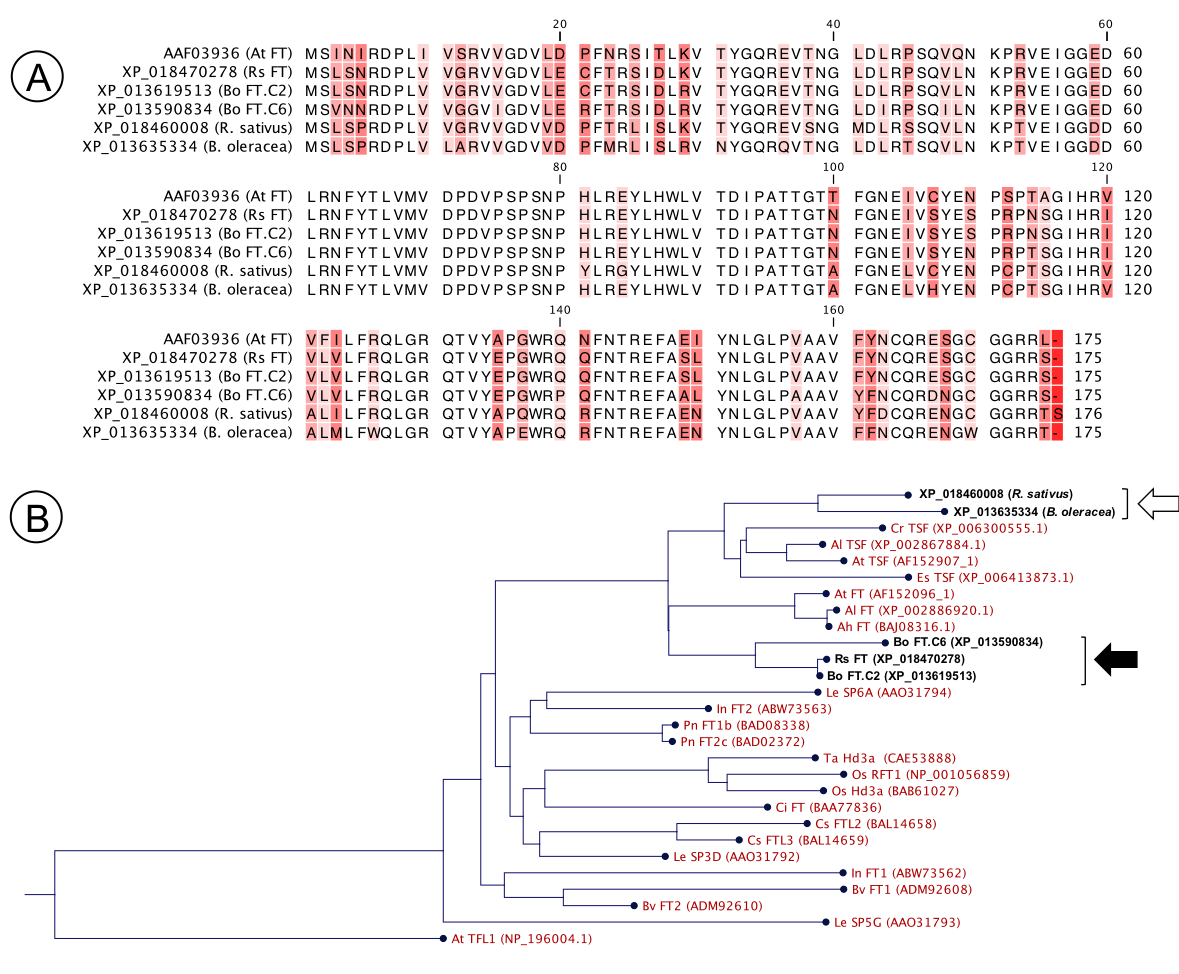


**Supplementary Figure 5.** FT homologs in *B. oleracea* and *R. sativus* gemome. **(A)** Alignment of amino acid sequence of *Arabidopsis* FT protein and FT–like proteins in *B. oleracea* and *R. sativus* genome. **(B)** A neighbor-joining phylogenetic tree of the FT gene family from several plant species, including FT-like genes of *B. oleracea* and *R. sativus*. The tree was constructed using the Neighbor-Joining method in CLC Sequence Viewer (https://www.qiagenbioinformatics.com/products/clc-sequence-viewer/). Black arrow and white arrow indicate the clades of FT and TSF genes, respectively, in *B. oleracea* and *R. sativus*. AtTFL1 was used as an outgroup. Ah, *Arabidopsis halleri*; Al, *Arabidopsis lyrata*; At, *Arabidopsis thaliana*; Bo, *Brassica oleracea*; Bv, *Beta vulgaris*; Ci, *Citrus unshiu*; Cr, *Capsella rubella*; Es, *Eutrema salsugineum*; Cs, *Chrysanthemum seticuspe*; In, *Ipomoea nil*; Le, *Lycopersicon esculentum*; Os, *Oryza sativa*; Pn, *Populus nigra*; Rs, *Raphanus sativus*; Ta, *Triticum aestivum*.

**
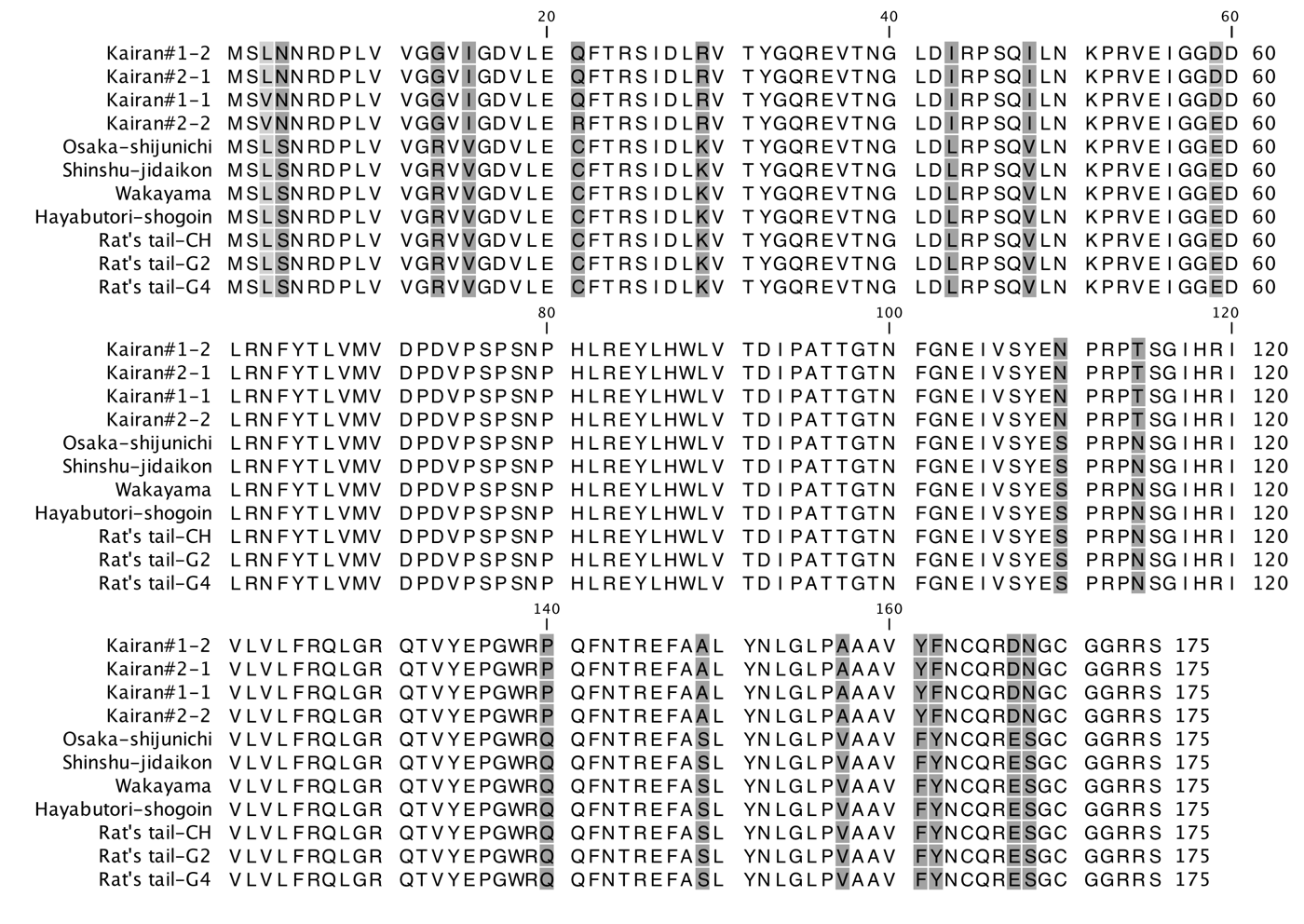
**

**Supplementary Figure 6.** Alignment of the *FT* homolog amino acid sequences of the rootstock plants used in the grafting experiments.

**
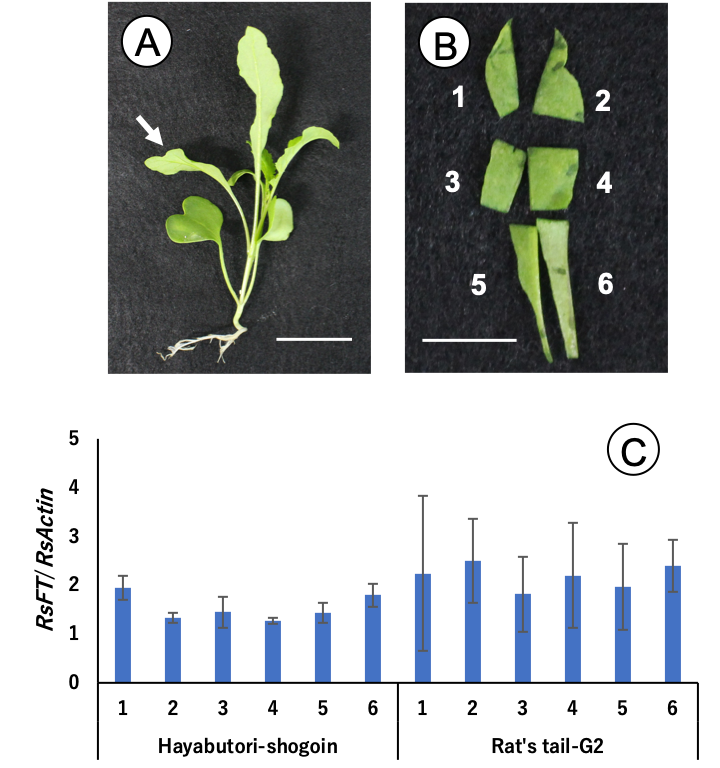
**

**Supplementary Figure 7.** Expression analysis of *RsFT* in the different part of the lowest leaves of the vernalized radish. The seeds of “Hayabutori-shogoin” and “Rat’s tail-G2” radish were vernalized at 2℃ for 7 weeks, and sown in a growth room maintained at 22 ± 2℃, under long-day (16/8, light (L)/dark (D)) conditions. At the day of bolting, the lowest leaves were collected at 1 h before the end of the light period. The sampled leaves whose petiole were removed were divided into six parts, and the expression level of *RsFT* for each part was determined by real-time RT-PCR. For the expression analysis, primer sets for *RsFT* and *RsActin* were used. **(A)** Typical bolting radish used for the analysis; the cultivar is “Rat’s tail-G2” radish. The white arrow indicates the lowest leaf. Bar = 3 cm. **(B)** The lowest leaf in (A) which was divided into six parts. Each part was numbered as shown in the picture. Bar = 1 cm. **(C)** Expression level of *RsFT*  in the different part of the lowest leaves. The numbers above the cultivar name indicate the position in the leaf shown in (B). For each cultivar, three plants were used for the analysis, except for position 5 in “Hayabutori-shogoin” and position 1 and 6 in “Rat’s tail-G2”, where only two plants were used because of the failure of RNA extraction. Error bars are the standard deviations of the mean. There was no significant difference in the expression levels of *RsFT* by the position within the leaf in both cultivars (one-way ANOVA, *p* > 0.05).

**
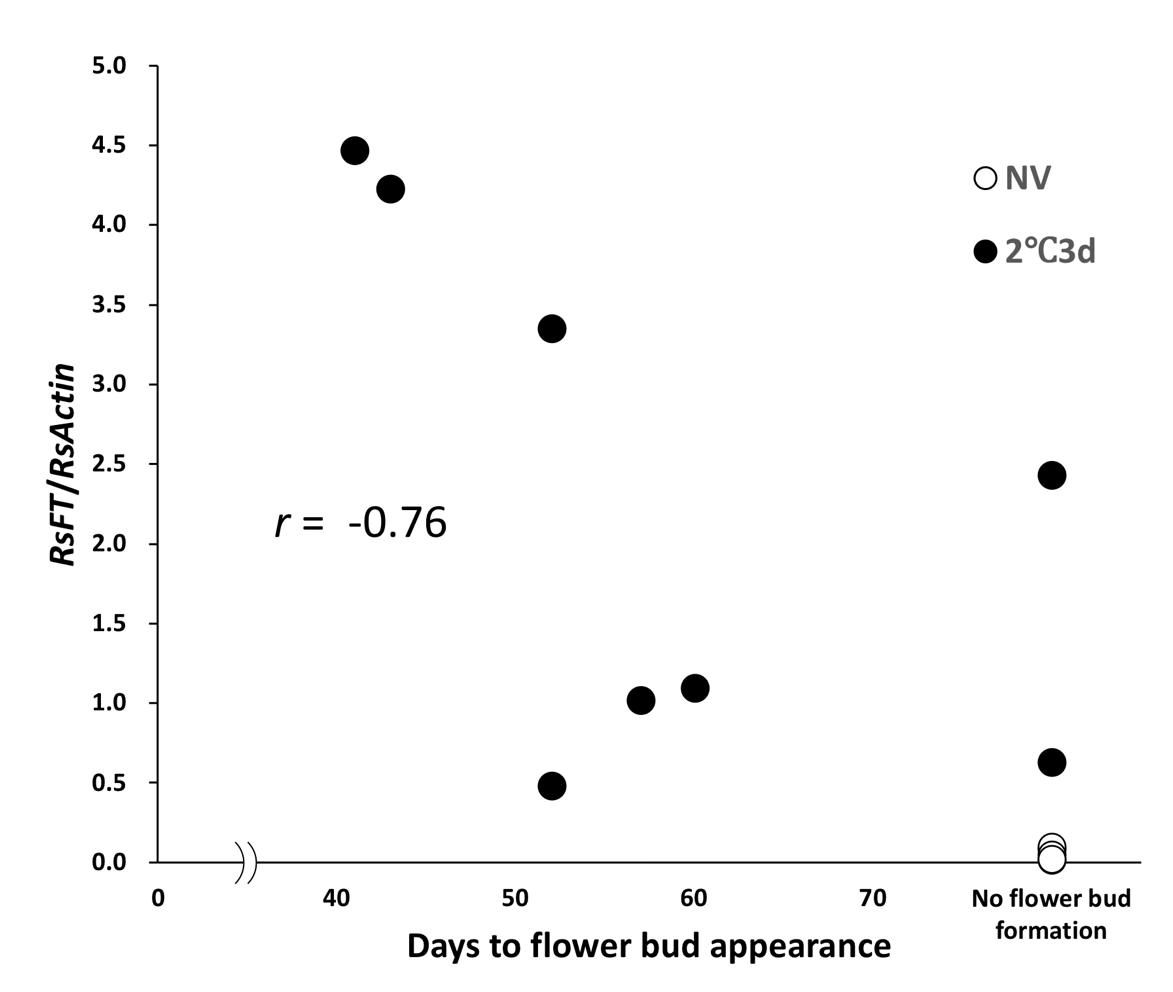
**

**Supplementary Figure 8.** The relationships between the relative expression level of *RsFT* in the lowest leaf of rootstock plants at 30 DAG and days to flower bud appearance in “Watanabe-seiko No.1” when grafted onto “Rat's tail-G2.” The scions that failed to develop flower buds until 60 DAG were given the score of 80 DAG for the calculation of the correlation coefficient. White circles (○) indicate the values for the non-vernalized “Rat's tail-G2” rootstock plants (*n* = 5), whereas black circles (●) indicate values for the 2°C 3 days vernalized “Rat's tail-G2” rootstock plants (*n* = 8).
